# On the ever‐growing functional versatility of the CRISPR‐Cas13 system

**DOI:** 10.1111/1751-7915.14418

**Published:** 2024-02-21

**Authors:** Roser Montagud‐Martínez, Rosa Márquez‐Costa, María Heras‐Hernández, Roswitha Dolcemascolo, Guillermo Rodrigo

**Affiliations:** ^1^ Institute for Integrative Systems Biology (I2SysBio) CSIC – University of Valencia Paterna Spain

## Abstract

CRISPR‐Cas systems evolved in prokaryotes to implement a powerful antiviral immune response as a result of sequence‐specific targeting by ribonucleoproteins. One of such systems consists of an RNA‐guided RNA endonuclease, known as CRISPR‐Cas13. In very recent years, this system is being repurposed in different ways in order to decipher and engineer gene expression programmes. Here, we discuss the functional versatility of the CRISPR‐Cas13 system, which includes the ability for RNA silencing, RNA editing, RNA tracking, nucleic acid detection and translation regulation. This functional palette makes the CRISPR‐Cas13 system a relevant tool in the broad field of systems and synthetic biology.

## Graphical abstract

The functional versatility of the CRISPR‐Cas13 system is discussed, which includes the ability for RNA silencing, RNA editing, RNA tracking, nucleic acid detection and translation regulation.

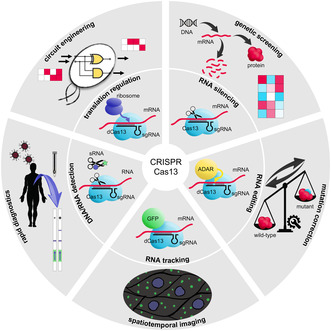

## INTRODUCTION

It was back in the 90s when the presence of clustered regularly interspaced short palindromic repeats (CRISPR) was first noticed within the genomes of halophilic archaea from the Spanish Mediterranean coast, providing early rough functional insight about such repeats (Mojica et al., 1995). At that time, however, it was hard to anticipate the revolution to come almost two decades later, starting with the ability to target and cleave DNA in a programmable way (Gasiunas et al., 2012; Jinek et al., 2012). CRISPR schemes evolved in many bacteria and archaea as adaptive immune systems in response to viral infections. Delightfully, these systems exploit ribonucleoproteins to combine the high programmability of RNA together with the strong stability and binding affinity of proteins. Thanks to genomic approaches, several CRISPR‐associated (Cas) proteins have been identified over the last years, which has ended with a classification that distinguishes two main classes and six types (Makarova et al., 2020).

**FIGURE 1:**
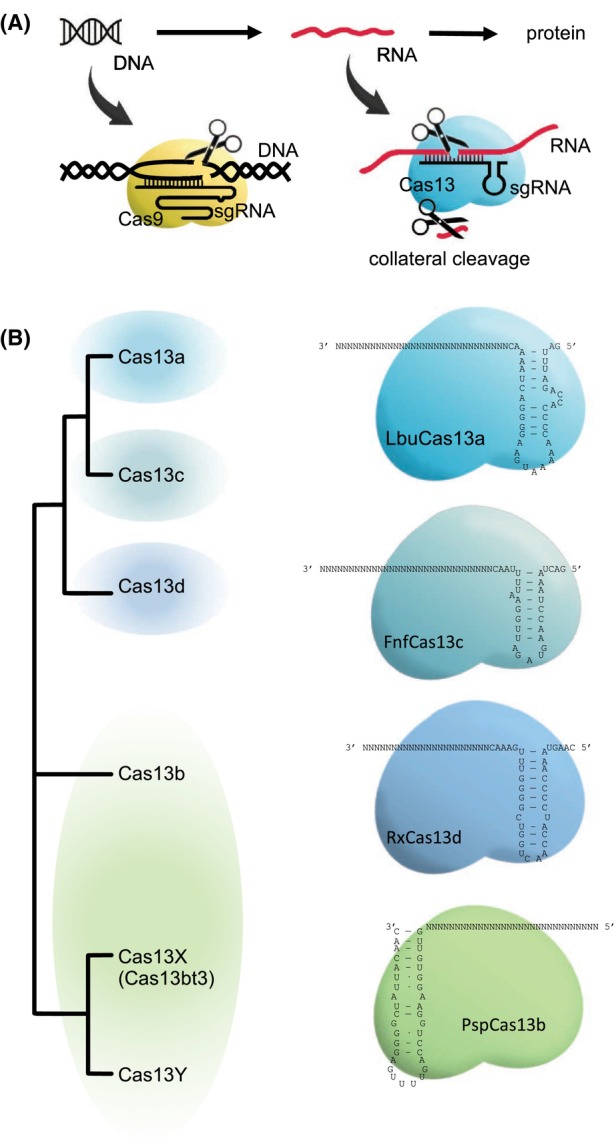
(A) Mechanistic overview of the CRISPR‐Cas9 and CRISPR‐Cas13 systems. (B) On the left, illustrative phylogenetic tree of the different Cas13 subtypes. On the right, representative sgRNAs for each subtype (sequence and structure). These are for *Leptotrichia buccalis* Cas13a (LbuCas13a), *Prevotella* sp. *P5‐125* Cas13b (PspCas13b), *Fusobacterium necrophorum* subsp. *funduliforme* Cas13c (FnfCas13c) and *Ruminococcus flavefaciens* Cas13d (RxCas13d).

**FIGURE 2:**
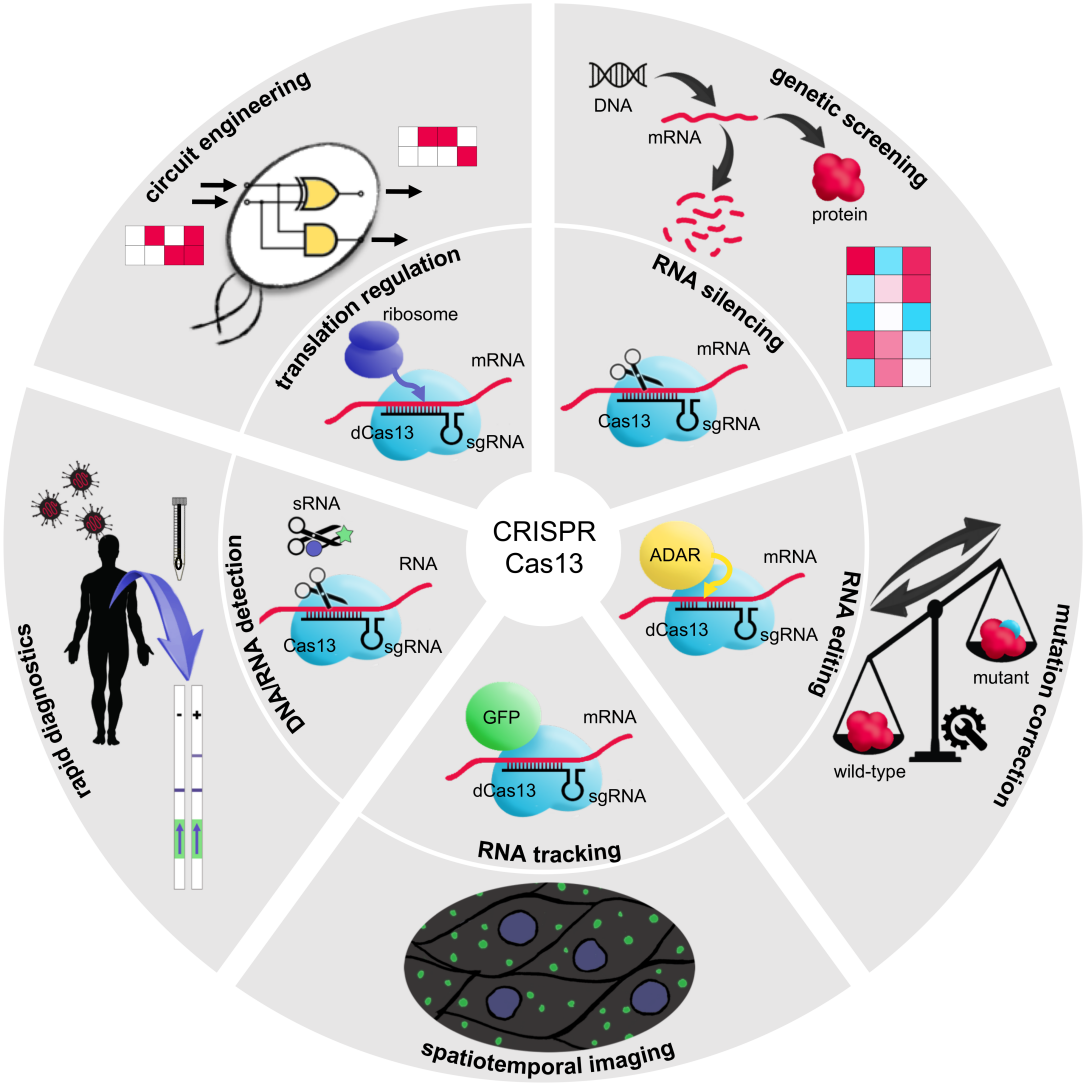
Schematics of the functional versatility of the CRISPR‐Cas13 system.

In this review, we focus on the CRISPR‐Cas13 system (class 2, type VI), in which an RNA‐guided RNA endonuclease (RNase) is at play (Abudayyeh et al., 2016; Shmakov et al., 2015). Figure 1A shows a mechanistic overview of the system. Importantly, Cas13 has two distinct RNase activities (East‐Seletsky et al., 2016). First, it is able to process its own small guide RNA (sgRNA) through a general acid–base catalysis mechanism (Mg^2+^‐independent). Second, it degrades the target RNA; thanks to two higher eukaryotes and prokaryotes nucleotide‐binding (HEPN) domains (Mg^2+^‐dependent; O'Connell, 2019). In addition, this RNase exhibits a collateral catalytic activity after target RNA recognition by which nearby RNA molecules are cleaved. There are four main subtypes according to the Cas proteins currently known: Cas13a, Cas13b, Cas13c and Cas13d (Figure 1B). Within the Cas13b type, Cas13X (also known as Cas13bt3) and Cas13Y have been revealed as particular subtypes. A recent study exploring metagenomic data has proposed additional subtypes according to the clades of the resulting phylogenetic tree (Hu et al., 2022). Certainly, their respective efficiency varies along different contexts. Cas13X and Cas13Y are the smallest proteins with about 775–800 amino acids in length, which contrasts with the about 1000–1200 amino acids of the rest (Xu et al., 2021). Moreover, there are fundamental differences in the cognate sgRNAs in terms of sequence and structure (e.g. the sgRNA mounted by Cas13a has the spacer in the 3′ end, while the sgRNA mounted by Cas13b in 5′; O'Connell, 2019). In very recent years, such CRISPR‐Cas13 systems have been repurposed for a series of applications beyond their natural function (Zhang, 2019). In the following, we present these advances in a concise manner by highlighting their relevance for the community of systems and synthetic biology, including the ability to regulate translation previously unnoticed (Figure 2). We also pay attention to the applicability of these systems in microbial biotechnology, from exploiting bacteria to fungi.

## CAS13 VERSUS CAS9

The CRISPR‐Cas9 system (class 2, type II) from *Streptococcus pyogenes* was first characterized and repurposed for biotechnological applications, exploiting its high modularity and efficiency (Jinek et al., 2012). This system is participated by an RNA‐guided DNA endonuclease (Cas9). Upon target site recognition, Cas9 induces a double‐stranded break in the DNA helix, initiating repair processes in the cell such as non‐homologous end joining (NHEJ) or homology‐directed repair (HDR; Xue & Greene, 2021). In stark, contrast to the CRISPR‐Cas13 system, an endogenous RNase III is recruited to process the sgRNA (Deltcheva et al., 2011) and Cas9 has no collateral activity because of an internal catalytic active site. The CRISPR‐Cas13 system appears as a complementary tool for biotechnology to target RNA instead of DNA and then have access to implementations at a different point in the genetic information flow to ensure genome integrity preservation, make them more transient and reversible and eventually have more flexibility to achieve orthogonality to the host cell machinery.

Following the programmable DNA targeting ability, the CRISPR‐Cas9 system has been exploited for genome engineering in a variety of organisms, including microbes (Jiang et al., 2013; Tong et al., 2021). Beyond HDR, which allows precise gene insertions or replacements by providing a template DNA alongside the sgRNA‐Cas9 complex, and NHEJ, which facilitates gene disruption through error‐prone repair mechanisms (Xue & Greene, 2021), base editing or prime editing with a Cas9 nickase (Cas9n) fused to a deaminase or a reverse transcriptase represent advanced techniques, enabling single‐base substitutions without inducing double‐strand breaks (Anzalone et al., 2019; Komor et al., 2016). The CRISPR‐Cas13 system can follow these developments to edit RNA (Cox et al., 2017; Jing et al., 2018). Beyond correcting deleterious mutations in the organism, this technology seems appropriate to induce transient responses to cope with stress. Moreover, the CRISPR‐Cas9 system has been exploited to regulate transcription initiation and elongation (Qi et al., 2013). The CRISPR‐Cas13 system complements this development by allowing either the silencing of specific transcripts or the regulation of translation initiation following the programmable RNA targeting ability (Abudayyeh et al., 2017; Montagud‐Martínez et al., 2023). Together, these versatile strategies are intended to engineer microbes with unprecedented precision, optimizing traits for industrial processes, bioenergy production and therapeutic applications.

## RNA SILENCING

Owing to its ability to target and cleave RNA, the CRISPR‐Cas13 system is a suitable tool to knock down gene expression at the posttranscriptional level. Importantly, some organisms have evolved Cas13 proteins able to work independently of particular sequence motifs in the target strand, such as the Cas13a from *Leptotrichia wadei* (formerly known as C2c2; Abudayyeh et al., 2017) or the Cas13d from *Ruminococcus flavefaciens* (formerly known as CasRx; Konermann et al., 2018). This makes the system well suited to target, in principle, any messenger RNA (mRNA). Yet, a local secondary structure in the mRNA may prevent an efficient binding with the ribonucleoprotein (Abudayyeh et al., 2016). Studies have shown that, in conducive sequence and structure conditions, the sgRNA‐Cas13 complex is able to knock down endogenous mRNAs, especially in eukaryotic contexts (Abudayyeh et al., 2017). In particular, Cas13d showed the greatest performance in human cells, even allowing the manipulation of alternative splicing (Konermann et al., 2018). Despite their small size, Cas13X and Cas13Y display a knock down efficiency comparable to Cas13d (Xu et al., 2021). However, the indiscriminate RNA degradation exerted by the complex upon targeting has been proven toxic for the host cell, especially in prokaryotic contexts but also in eukaryotes (Ai et al., 2022). This concern has prevented its use for synthetic posttranscriptional gene silencing in a reliable way. In this regard, engineering efforts to obtain variants with significant RNA knock down activity and marginal collateral damage are appealing (Tong et al., 2023). Another issue comes from the cell‐dependent activity of the ribonucleoproteins to target RNA (e.g. working much better in mammalian cells than in yeast; Zhang et al., 2022), thereby limiting the dynamic range of the responses (especially in bacteria, where the competition with ribosomes is fierce; Montagud‐Martínez et al., 2023) and preventing an orthogonal deployment across phyla.

This technology may enable large‐scale genetic screening aimed at discovering natural protein function, at the same time computational descriptors of sgRNA activity are developed (Wessels et al., 2020). Traditionally, gene knock down in higher eukaryotes, such as mammals or plants, has been carried out by exploiting the endogenous RNA silencing machinery (RNA interference; Wilson & Doudna, 2013) through designer small RNAs (sRNAs) able to fold into hairpins that mimic the expression of natural elements (Schwab et al., 2006; Siolas et al., 2005). The CRISPR‐Cas13 system may achieve comparable repression folds and off‐target effects (Abudayyeh et al., 2017; Konermann et al., 2018). In prokaryotes, by contrast (in absence of RNA interference), posttranscriptional control of gene expression has typically been accomplished through synthetic sRNAs specially designed to block translation initiation (Ghodasara & Voigt, 2017). In bacteria, a controlled expression (then toxicity) of Cas13d through a low copy number vector, a tightly regulated promoter and a weak ribosome binding site allowed its use to repress gene expression (Zhang et al., 2020). Furthermore, the CRISPR‐Cas13d system was harnessed to induce RNA silencing in fission yeast using a CRISPR array (Chen et al., 2023).

At this point, it is also important to note that RNA targeting can be achieved with other CRISPR systems. Recently, the CRISPR‐Csm system (class 1, type III), which relies on an effector complex of five proteins (Csm1‐5) and uses the endonuclease Cas6 for sgRNA maturation, has been programmed to degrade RNA with high efficiency and minimal off‐target effects (Colognori et al., 2023). The CRISPR‐Cas7‐11 system (class 1, type III), using a single protein effector, has been engineered for RNA knock down too (Özcan et al., 2021). In addition, it has been shown that some Cas9s are able to target RNA in a protospacer adjacent motif (PAM)‐independent way (Strutt et al., 2018; Zhang et al., 2022). Other endonucleases, such as Cas6f (class 1, type I; formerly known as Csy4) or Cas12a, have been used to cleave a specific mRNA and induce its degradation (Qi et al., 2012; Yu & Marchisio, 2023), although not in a programmable way (i.e. through genetic modification of the target). We see these developments complementary to the CRISPR‐Cas13 system to meet all requirements of the RNA targeting applications.

## ANTIVIRAL ACTIVITY

CRISPR‐Cas13 systems evolved in bacteria and archaea to fend off viral attacks. Importantly, it has been shown that this immune system is able to protect against both RNA and DNA phages as a result of the combined ability to degrade viral RNA in a guided way and host transcripts in a collateral way (Meeske et al., 2019). From a biotechnological perspective, it has been possible to implement a synthetic protection in *Escherichia coli* against phages (Adler et al., 2022), as well as to limit the intracellular replication of RNA viruses that infect humans or plants (Aman et al., 2018; Freije et al., 2019), showing exciting clinical and agricultural prospects. In addition, exploiting this antiviral activity for counterselection in bacteria, the genomes of diverse phages were edited without leaving a trace (Adler et al., 2022). This development seems relevant because phage engineering represents a promising weapon in the ongoing battle against antibiotic‐resistant bacteria (Khambhati et al., 2023). The use of phages allows for the precise targeting of pathogenic bacteria, while minimizing collateral damage to beneficial microorganisms. Collectively, as our understanding of this RNA targeting system continues to expand, we can anticipate further breakthroughs in the fight against infectious diseases, ultimately improving public health and safety.

## RNA EDITING

Cas proteins can be appropriately mutated to abolish their catalytic activity. This is intended to exploit the targeting without cleaving of nucleic acids to take molecular machinery to specific loci. In particular, a catalytically dead Cas13 (dCas13) is obtained by mutating the HEPN domains, which prevents the target RNA degradation but maintains the sgRNA processing (Abudayyeh et al., 2017; East‐Seletsky et al., 2016). Thereby, an immediate application consists in editing the genetic code. The CRISPR‐Cas9 system was first repurposed for precise genome editing by fusing a cytidine deaminase to dCas9 (Komor et al., 2016), which was later expanded thanks to using adenine base editors obtained by directed evolution (Gaudelli et al., 2017). Alternatively, the CRISPR‐Cas13 system is a suitable tool for editing the genetic code at the RNA level. Importantly, this enables an alteration of protein function that is reversible and with no risk of creating permanent off‐target mutations in the genome. Indeed, RNA can be edited in a precise way by fusing an adenosine deaminase to dCas13 (Cox et al., 2017; Jing et al., 2018). In detail, the editing site (adenosine) is defined by a mismatch in the spacer of the sgRNA, which leads to the conversion of the adenosine residue into inosine. This new residue is read as a guanosine by the translation machinery, which in effective terms is like an A‐to‐G edit (Nishikura, 2016). To enlarge the repertoire of possible phenotypic mutations, an adenosine deaminase was appropriately mutated and selected to achieve C‐to‐U edits (i.e. conversion of that natural enzyme into a cytidine deaminase; Abudayyeh et al., 2019).

The functional consequences of the edit depend on its position on the RNA molecule (mostly mRNA). A single amino acid exchange from an edited mRNA may lead to a protein product with altered structure and function (Kato et al., 2003). Moreover, the open reading frame may be changed upon modifying start or stop codons, or even splicing signals (Nishikura, 2016). In addition to effects only revealed posttranslationally, the introduction of inosine may affect RNA folding (and then RNA half‐life), RNA processing of regulatory sRNAs, RNA transport and RNA–RNA or RNA–protein interactions (Shevchenko & Morris, 2018). It is worth noting, however, that there are alternatives to the CRISPR‐Cas13 system to efficiently edit RNA in a programmable way, such as recruiting endogenous deaminases with engineered RNA hairpins and even circular RNAs (Merkle et al., 2019; Yi et al., 2022) or targeted pseudouridylation through H/ACA‐box small ribonucleoproteins (Karijolich & Yu, 2011), with this last approach successfully applied in yeast. Beyond base editing, the programmed *N* ^6^‐methyladenosine modification of mRNA with a dCas13‐directed methyltransferase has also been achieved, with notable effects on expression (Wilson et al., 2020).

In bacteria, RNA editing is usually precluded from biotechnological efforts due to the high unstability of the mRNA molecules (about 5 min of half‐life; Bernstein et al., 2002), as a result of the tight action of the endogenous RNases. Besides, research was conducted to harness a Cas13a to promote the edition of endogenous and exogenous transcripts in *Schizosaccharomyces pombe* (Jing et al., 2018). Such advances in RNA editing and silencing may lead to new metabolic control implementations in yeast for the efficient bioproduction of compounds of clinical and industrial importance (Wang et al., 2023). Overall, CRISPR‐Cas13‐mediated RNA editing provides a promising opportunity to exert therapeutic actions aimed at correcting undesired mutations and to engineer synthetic gene circuits with sophisticated posttranscriptional regulations.

## RNA TRACKING

RNA spatiotemporal dynamics is increasingly being recognized as an important determinant of gene expression, function and regulation (Kannaiah et al., 2019; Nowakowski et al., 2017). Thus, methods for monitoring mRNAs or sRNAs among tissues, cells and even subcellular compartments are required. In turn, the ability to track RNA in vivo can also be instrumental to study the within‐host propagation of viral infections (Tromas et al., 2014). Roughly, previous methods for RNA imaging consisted in either chemically synthesized oligonucleotides appropriately labelled with fluorescent dyes to be delivered into cells (e.g. fluorescent in situ hybridization) or suitable aptamers tagging the transcript of study to be recognized by RNA‐binding proteins fused to fluorescent proteins (Crosetto et al., 2015; Tyagi, 2009). The former approach has the enormous advantage of not requiring the manipulation of the RNA of study, which limits interferences in natural function, while the latter the benefit of being fully genetically encodable, which allows greater spatiotemporal resolution. Recently, inspired by the use of the CRISPR‐Cas9 system to image DNA loci (Chen et al., 2013), the CRISPR‐Cas13 system with a catalytically dead protein has been exploited to image RNAs in living cells (Abudayyeh et al., 2017), allowing a dynamic characterization without genetic modification. In particular, transcripts can be tracked after having fused a fluorescent protein to dCas13 (Abudayyeh et al., 2017; Yang et al., 2019) or by using sgRNAs labelled with fluorescent dyes (for delivery‐based approaches only; Wang et al., 2019). Alternatively, the CRISPR‐Cas9 system was also repurposed to track RNA, although at the cost of providing an additional oligonucleotide (Nelles et al., 2016). The CRISPR‐Csm system (with catalytically inactive Csm) was also exploited to image RNA thanks to a specific and durable binding (Colognori et al., 2023). To enhance the resolution in the case of low concentration, multiple sgRNAs targeting the same transcript may be designed or, instead, the sgRNA may be modified to accommodate several aptamers (e.g. the kind aptamers aforementioned or even aptamers that can fluoresce in response to a ligand; Ma et al., 2018). Yet, this latter strategy still needs to be proved in the case of the CRISPR‐Cas13 system. Furthermore, a dCas13 was fused to an engineered peroxidase to check protein–RNA interactions through protein biotinylation coupled to mass spectrometry (Han et al., 2020). Certainly, as our ability to monitor RNA in vivo increases, different biological phenomena of outstanding dynamic nature, such as transcriptional bursting or intercellular gene expression variability (Raj & van Oudenaarden, 2009), will be better and more broadly appreciated.

## NUCLEIC ACID DETECTION

Due to the ability of some Cas proteins to display a collateral catalytic activity (as a result of strand release upon cleavage), CRISPR‐Cas systems have also been repurposed for rapid, specific and portable nucleic acid detection (Li et al., 2019). Currently, the standard diagnostic technique is based on polymerase chain reaction (PCR) and variants of it (e.g. with a reverse transcription step to detect RNA instead of DNA or with a quantitative protocol to determine the concentration in the sample). This has allowed the identification of different pathogenic microorganisms in clinical and environmental samples (Call et al., 2003; Yang & Rothman, 2004) and, together with sequencing techniques, the disclosure of relevant mutations that have enable more accurate prognoses and epidemiological reconstructions (Kao et al., 2014). Yet, isothermal amplification methods (e.g. recombinase polymerase amplification) represent a suitable alternative to speed up the process and bypass the need of precise equipment (Zhao et al., 2015). CRISPR‐Cas systems emerge at this point as opportune molecular tools to discriminate the amplified sequences, avoiding false positives and then produce a readable output signal. Remarkably, after an additional step of in vitro transcription, an appropriately programmed sgRNA‐Cas13 complex can cleave its RNA target and subsequently a sRNA probe appropriately labelled in its two ends (e.g. fluorophore‐quencher for fluorometry or fluorophore‐biotin for colorimetry; Gootenberg et al., 2017, 2018). Alternatively, the CRISPR‐Cas12 system (participated by an RNA‐guided DNA endonuclease; class 2, type V) has also been proven effective to detect nucleic acids by targeting amplified DNA fragments and exerting a collateral activity on single‐stranded DNA (ssDNA) molecules, bypassing the need for the in vitro transcription (Chen et al., 2018). Surprisingly, some Cas12 nucleases, such as Cas12a2 or Cas12g, have demonstrated an intriguing ability to target RNA followed by a *trans*‐cleavage activity on both sRNA and ssDNA molecules (Bravo et al., 2023; Dmytrenko et al., 2023; Yan et al., 2019), with Cas12a2 also *trans*‐cleaving dsDNA molecules, which highlights the plasticity of the CRISPR‐Cas systems and prompts different nucleic acid detection strategies.

Importantly, the resulting systems exhibit great concentration sensitivity (at the attomolar scale, about one molecule per microlitre of sample; Gootenberg et al., 2017) and high sequence specificity (even at one nucleotide resolution, as they are sensitive to mismatches in the spacer of the sgRNA or modifications of the PAM). In particular, the CRISPR‐Cas13 system was already used with success to detect human‐infecting RNA viruses (Myhrvold et al., 2018) and biomarker‐acting microRNAs (Shan et al., 2019), and even to complement enzyme‐linked immunosorbent assays for enhanced protein detection (Chen et al., 2020). Of note, the Cas13a from *Leptotrichia buccalis* displays the greatest collateral activity (East‐Seletsky et al., 2017), which has led to its exploitation to detect the novel coronavirus RNA genome even without pre‐amplification (Fozouni et al., 2021). Moreover, the use of multiple sgRNAs working in parallel (i.e. targeting different regions of the RNA molecule of interest) can increase the sensitivity and specificity of the detection, and the combination of different nucleases, such as Cas13a and Csm6, allows enhancing the detectability as a result of a sequential *trans*‐cleavage of two singular sRNA probes (Liu et al., 2021). In this regard, we anticipate that CRISPR‐Cas systems will have an important impact on clinical diagnostics and also on environmental monitoring. This technology paves the way not only to provide point‐of‐care solutions but to develop, together with compact nanotechnological devices (e.g. for lateral flow immunochromatographic assays; Yetisen et al., 2013), simple self‐diagnostic tests.

## TRANSLATION REGULATION

The ability to regulate at different points of the genetic information flow is key to construct increasingly complex gene expression programmes. In this regard, the development of novel posttranscriptional mechanisms is appealing. The ability of CRISPR‐dCas13 ribonucleoproteins to target RNA in a programmable way and without degradation can be exploited to regulate translation. Recently, such a framework has been developed using the dCas13a from *L. wadei* (dead version) in a cell‐free expression system for prototyping gene regulatory structures (Montagud‐Martínez et al., 2023). Those results demonstrate that these ribonucleoproteins can be programmed to repress or activate translation initiation. In particular, activation was accomplished by inducing a conformational change in the 5′ untranslated region (UTR) to release the ribosome binding site, following a mechanism previously demonstrated with sRNAs (Isaacs et al., 2004). This capability of dCas13a was also confirmed in *E. coli* (Montagud‐Martínez et al., 2023), although more work is required in this direction to enhance the dynamic regulatory ranges and engineer functional gene circuits. Mostly in prokaryotic systems, translation regulation is location‐dependent due to tight sequence determinants of initiation (Espah‐Borujeni et al., 2014). Translation activation can additionally be achieved by recruiting endogenous translations factors. The dCas13d from *R. flavefaciens* was fused to a translation initiation factor to enhance protein expression in *E. coli* (Otoupal et al., 2022). Alternatively, the sgRNA associating with dCas13d was fused to an RNA domain known to promote ribosome recruitment for increasing protein expression in human cells (Cao et al., 2023). In addition, CRISPR‐dCas13 ribonucleoproteins were used to target endogenous sRNAs in *E. coli* and then change the translation rate of cognate genes, a strategy that was further exploited to increase the production of lycopene (Ko & Woo, 2023). These developments complement the ability to regulate transcription with other CRISPR‐Cas systems and offer potential applications. The ability to regulate translation may allow intra‐operon regulation in bacteria (i.e. the control of a specific gene from a polycistron; Balasubramanian & Vanderpool, 2013), with implications for metabolic engineering (Pfleger et al., 2006). Moreover, this new ability may facilitate characterizing RNAs with dual function (i.e. coding for a protein and interacting with further RNAs for regulatory purposes; Ulveling et al., 2011). In biomedicine, this would hold significance because the perturbation of such a duality in key genes may lead to important diseases in humans (Poliseno et al., 2010). In contrast to the RNA silencing functionality, the toxicity to the host cell in the case of translation control is alleviated due to the use of dCas13 with no or marginal collateral activity.

## CONCLUSION

The programmable nature of the CRISPR ribonucleoproteins to target any DNA or RNA sequence, provided some sequence and structure requirements are met, is being successfully exploited to repurpose these elements for a variety of applications (Table 1). In combination with CRISPR‐Cas9/Cas12 systems (DNA targeting), we anticipate that the functional versatility here presented of the CRISPR‐Cas13 system (RNA targeting) will allow, on the one hand, the discovery at a further level of natural gene function and the establishment of links between genotypes and phenotypes (systems biology). On the other hand, it will boost the engineering of novel synthetic gene circuits to (re)programme living cells (synthetic biology). As the CRISPR‐Cas13 system demonstrates potential in microbial biotechnology, therapeutic and diagnostic applications in humans and animals may expand. Bacterial cells or viruses can be precisely engineered to exert therapeutic actions in the body (from treating infectious diseases to genetic diseases such as cancer; Chowdhury et al., 2019; Mazzolini et al., 2023) and, in this regard, the CRISPR‐Cas13 system could be employed as a tool for such genome engineering (e.g. phage engineering; Adler et al., 2022) or as a programmable genetic module to implement new regulations in those genomes (Wang et al., 2024). In agriculture, viral vectors carrying CRISPR systems could be instrumental to modify crop traits by regulating specific plant genes (Yu et al., 2020), with the aim of optimizing yields and resilience and ultimately contributing to global food security and sustainability. Future work should also explore the use of the CRISPR‐Cas13 system to control engineered metabolic pathways for bioproduction or biodegradation (Ko & Woo, 2023). Indeed, a posttranscriptional control of enzyme expression in microbes has been shown to be a highly effective and versatile strategy to fine‐tune metabolic capabilities (Na et al., 2013). However, despite its potential, there are still challenges that need to be overcome in order to fully realize the potential of the CRISPR‐Cas13 system. The apparent toxicity of the collateral catalytic activity in vivo seems the main drawback, but the use of engineered versions may alleviate this issue. Moreover, other RNA targeting systems can complement the CRISPR‐Cas13 system to develop an increased ability to engineer gene regulation, gene editing and nucleic acid detection and tracking. Either for the genetic (re)engineering of bioproduction cells in the industry or for performing therapeutic or diagnostic actions in the clinic, CRISPR‐based RNA targeting could have a remarkable impact in the coming years.

**TABLE 1:** Summary of principal applications achieved with CRISPR‐Cas13 systems in or with microbes.

System	Application	Environment	Performance	Reference
CRISPR‐Cas13a	RNA silencing	Bacteria	Repression about 50%	Abudayyeh et al. (2017)
CRISPR‐Cas13d	RNA silencing	Bacteria	Repression about 50%	Zhang et al. (2020)
CRISPR‐Cas13d	RNA silencing	Fission yeast	Repression about 80%	Chen et al. (2023)
CRISPR‐Cas13a	Phage engineering	Bacteria	Markerless genome edits with 100% efficiency	Adler et al. (2022)
CRISPR‐dCas13a	RNA editing	Fission yeast	Editing efficiency up to 50%	Jing et al. (2018)
CRISPR‐Cas13a	Diagnostics	In vitro	Human virus detection with up to 1 aM sensitivity	Gootenberg et al. (2017)
CRISPR‐Cas13a + CRISPR‐Cas13b	Diagnostics	In vitro	Multiplexed human virus detection	Gootenberg et al. (2018)
CRISPR‐Cas13a	Diagnostics	In vitro	Human virus detection without pre‐amplification with up to 0.1 fM sensitivity	Fozouni et al. (2021)
CRISPR‐dCas13a	Repression and activation of translation initiation	Cell‐free system and bacteria	Repression up to 80% and activation up to 200%	Montagud‐Martínez et al. (2023)
CRISPR‐dCas13d	Enhancement of protein expression	Bacteria	Enhancement up to 20‐fold	Otoupal et al. (2022)
CRISPR‐dCas13a	Repression of sRNAs	Bacteria	Repression up to 90% and bioproduction increase up to fourfold	Ko and Woo (2023)

## AUTHOR CONTRIBUTIONS

**Roser Montagud‐Martínez:** Visualization (lead); writing – original draft (equal). **Rosa Márquez‐Costa:** Writing – original draft (equal). **María Heras‐Hernández:** Writing – original draft (equal). **Roswitha Dolcemascolo:** Writing – original draft (equal). **Guillermo Rodrigo:** Conceptualization (lead); funding acquisition (lead); visualization (supporting); writing – original draft (equal).

## CONFLICT OF INTEREST STATEMENT

The authors declare no financial or commercial conflict of interest.
